# Expression of cell proliferation and apoptosis biomarkers in pterygia and normal conjunctiva

**Published:** 2011-06-23

**Authors:** Kun Liang, Zhengxuan Jiang, Bi-qing Ding, Ping Cheng, Da-ke Huang, Li-ming Tao

**Affiliations:** 1 Department of Ophthalmology, the Second Hospital Affiliated to Anhui Medical University, Hefei, Anhui, P.R. China; 2 Department of Pathogenic Biology, College of Basic Medicine, Anhui Medical University, Hefei, Anhui, P.R. China

## Abstract

**Purpose:**

To analyze the expression of apoptosis and cell proliferation molecules in pterygium tissues of Chinese patients.

**Methods:**

Thirty-three pterygia were surgically removed using the bare sclera procedure, and 23 normal bulbar conjunctivas were also obtained. Formalin-fixed, paraffin-wax-embedded tissues were analyzed by immunohistochemistry with anti- proliferating cell nuclear antigen (PCNA), K_i_-67 (a proliferating cell marker), mutant p53 (mP53), Bcl-2 associated X-protein (BAX), B-cell lymphoma gene 2 (Bcl-2), and caspase-3 antibodies. Terminal deoxynucleotidyl transferase-mediated dUTP-biotin nick end labeling assay (TUNEL) analysis was used to analyze the apoptotic cells.

**Results:**

Our study revealed that the positive rate of PCNA and K_i_-67 significantly increased in the pterygium samples compared to the normal conjunctiva samples. In the molecules involved in apoptosis, the results showed that the positive rate of Bcl-2 and mP53 significantly increased in the pterygium samples. However, no difference was found between the pterygium and normal conjunctiva samples in the expression of Bax and caspase-3. Through TUNEL analysis, apoptotic cells were seen in the entire width of the epithelial layer in normal conjunctivas but were found mainly confined to the outer layer of the epithelial cells in pterygia.

**Conclusions:**

The finding of high levels of cellular proliferation and low levels of cellular apoptosis in pterygia confirmed that both cell apoptosis and proliferation are known to play an important role in human pterygium pathogenesis.

## Introduction

Pterygium is one of the most commonly seen diseases in ophthalmology. It is a fibrovascular neoformation characterized by a triangular or wing-shaped overgrowth of abnormal conjunctiva onto the cornea and is composed of epithelium and highly vascular, subepithelial, loose connective tissue [1]. In severe cases, a pterygium can grow into the central cornea, which can induce irregular corneal astigmatism, resulting in loss of vision [2]. It frequently recurs after resection.

The pathogenesis of pterygia has not yet been clarified. Pterygia show significant differences from bulbar conjunctivas, both in the epithelium and in the underlying connective tissue [3]. Pterygia share many similar traits with tumors, such as cell proliferation, invasion of the cornea, and recurrence after resection [4]. Recurrent pterygium is more common in younger patients and is often associated with a family history of pterygium. It also requires sophisticated surgery [5]. Recently, pterygium has been thought to be an uncontrolled cell proliferation [6]. Kase et al. [7] showed that pterygium growth and development were associated with the proliferation of the epithelium. Other researchers have determined that pterygium is a disorder of excessive cellular proliferation in the fibrovascular layer [8]. Still others have shown similar cellular proliferation patterns between pterygia and conjunctivas [9]. Tan et al. [10] examined pterygium specimens for the pattern of expression of genes known to be involved in the regulation of apoptosis, and they drew the conclusion that pterygia were a result of disruption of the normal process of apoptosis. We designed the current study to determine whether cellular proliferation or/and cellular apoptosis participate in the pathogenesis of pterygia.

In this study, we examined the expression biomarkers of proliferation (proliferating cell nuclear antigen [PCNA], K_i_-67 [a proliferating cell marker]) and apoptosis (Bcl-2 associated X-protein [BAX], B-cell lymphoma gene 2 [Bcl-2], caspase-3, and mutant p53 [mP53]) in pterygia and normal conjunctivas by immunohistochemistry. We also directly observed apoptotic cells by TUNEL (terminal deoxynucleotidyl transferase-mediated dUTP nick end labeling) analysis.

## Methods

### Patients and controls

Informed consent was obtained from all individuals who participated in this study. The study group included 33 cases of surgically excised pterygium (19 males and 14 females) with an age range of 43–79 years and an average age of 66.3 years. All patients underwent excision by the bare sclera technique. The normal group comprised 23 individuals (12 males and 11 females) with an age range of 47–81 and an average age of 66.9 who had other eye diseases but exhibited no characteristics of pterygium. Normal conjunctiva samples were collected from the superior conjunctivas of the normal group individuals during their cataract or vitreoretinal surgery. All patients underwent a complete ophthalmic examination.

### Immunohistochemistry study

All sections were deparaffinized in xylene, sequentially rehydrated in alcohol, and washed in phosphate-buffered saline. The sections were heated twice in a microwave oven for 5 min in citrate buffer (pH 6.0) for antigen retrieval. Anti-PCNA monoclonal antibody (1:300; Santa Cruz Biotechnology Inc., Santa Cruz, CA), rabbit polyclonal anti-K_i_-67 antibody (prediluted; Zhongshan biologic and technical company, Beijing, China), rabbit polyclonal anti-Bax (p19), anti-Bcl-2 (c21) antibody (1:150; Santa Cruz), anti-caspase-3 (1:70; Zhongshan biologic and technical company), and anti mutant p53 (1:400; Santa Cruz) were used as the primary antibody. Briefly, the incubation was at 4 °C overnight, followed by washing with PBS. The sections were incubated with secondary antibody for 30 min at room temperature. Signals were developed with 3,3’-diaminobenzine (DAB) for 5 min and counterstained with hematoxylin [11,12]. Negative controls were performed with normal mouse or rabbit sera diluted at the same concentration as the primary antibody.

### TUNEL analysis

For TUNEL staining, tissue sections were deparaffinized, rehydrated through a series of graded alcohols, and washed in distilled water followed by PBS. Subsequently, the sections were permeabilized using proteinase K (20μg/ml, 20 min at room temperature), washed in PBS, incubated in 2% H_2_O_2_ in 0.1 M PBS for 30 min at room temperature to quench endogenous peroxidase activity, and treated with Triton-X. The terminal deoxynucleotidyl transferase-mediated dUTP nick end labeling (TUNEL) reaction was performed using a cell death kit (Roche, Mannheim, Germany). Briefly, the slides were incubated with TUNEL reaction mixture for 60 min (humid chamber, 37 °C) and then washed twice in PBS. After multiple washing steps, the sections were treated with Converter-POD solution for 30 min (humid chamber, 37 °C), rinsed with PBS, and visualized by adding 3,3′-diaminobenzine (DAB) for 10 min at room temperature. They were then washed in phosphate buffer saline (PBS), counterstained using hematoxylin staining, and finally, mounted for light microscopic observation. For negative controls, sections were incubated with label solution only (without terminal transferase) instead of the TUNEL reaction mixture.

### K_i_-67 positive cell counting

All pterygium basal epithelial cells were counted and positive staining cells of five microscopic fields (400×) from each tissue was performed. Then, positive staining cells were noted by their labeling index as a percentage in each specimen, and the measurements were averaged [13].

### Statistical analysis

SPSS (the Statistical Package for Social Sciences) 13.0 (SPSS Inc., Chicago, IL) was used for data analysis, and p<0.05 was considered to be significant. Wilcoxon rank sum test and χ^2^ test were used to detect the data. When the qualification of χ^2^ test wasn't satisfied, continuity correlation was used to modify the results.

## Results

In this study, we tested the expression of the proliferation-related genes *K_i_-67* and *PCNA* and the apoptosis genes *p53*, *Bcl-2*, and *Bax* in 33 samples of pterygia and 23 samples of normal conjunctivas. The results are shown in Table 1. TUNEL methodology was used to test the evidence of apoptotic cells.

**Table 1:** Number of positive and negative results for cell proliferation and apoptosis biomarkers in pterygium and conjunctiva samples.

** **	**Pterygium samples**		**Conjunctiva samples**		** **
** Factor**	**+**	**-**	**+**	**-**	**p value**
PCNA	24	9	7	16	0.002
K_i_-67	33	0	23	0	–
mP53	15	18	0	23	0.001
Bcl-2	33	0	2	21	0.000
Bax	33	0	23	0	–
Caspas-3	14	19	13	10	0.299

+:positive;-: negative; p value: the positive rate of biomarkers in pterygia versus in the conjunctivas.

**Figure 1:**
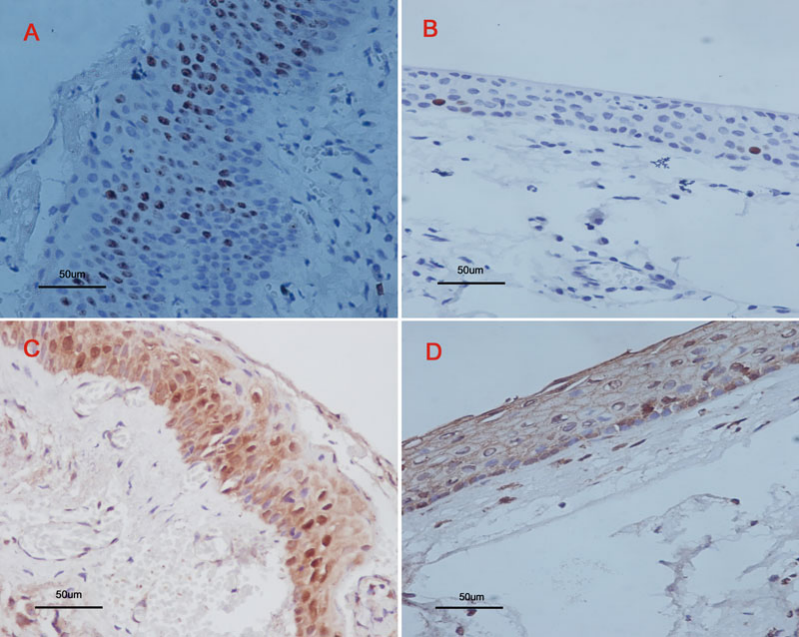
Immunohistochemical staining for K_i_-67 and PCNA positive cells in pterygium and normal conjunctiva samples. Positive K_i_-67 immunostaining showed higher nuclear staining in pterygia (**A**) than in normal conjunctivas (**B**). PCNA immunostaining showed a higher nuclear staining in pterygia (**C**) than in normal conjunctivas (**D**). All slides were counterstained with Mayer’s hematoxylin. Original magnifications: **A**-**D**, 400×.

K_i_-67 protein expression was observed in all pterygium cases and all normal conjunctiva cases. However, the number of immunopositive cells in the epithelial layer of pterygia (14.27±4.43) was significantly higher than that in normal conjunctivas (4.26±2.42, p<0.01; Table 2, Figure 1A,B). PCNA-positive immunostaining was evaluated in 24 of the 33 cases of pterygia and 7 of the 23 cases of normal conjunctiva. The positive rate of PCNA was significantly higher in pterygia than in normal conjunctivas (p=0.002; Table 1, Figure 1C,D).

**Table 2:** Number of cells positive for K_i_-67 in the basal epithelium of pterygium and conjunctiva samples.

**K_i_-67**	**Pterygium (%)**	**Conjunctiva (%)**
±S	14.27±4.43	4.26±2.42

p<0.01.

**Figure 2:**
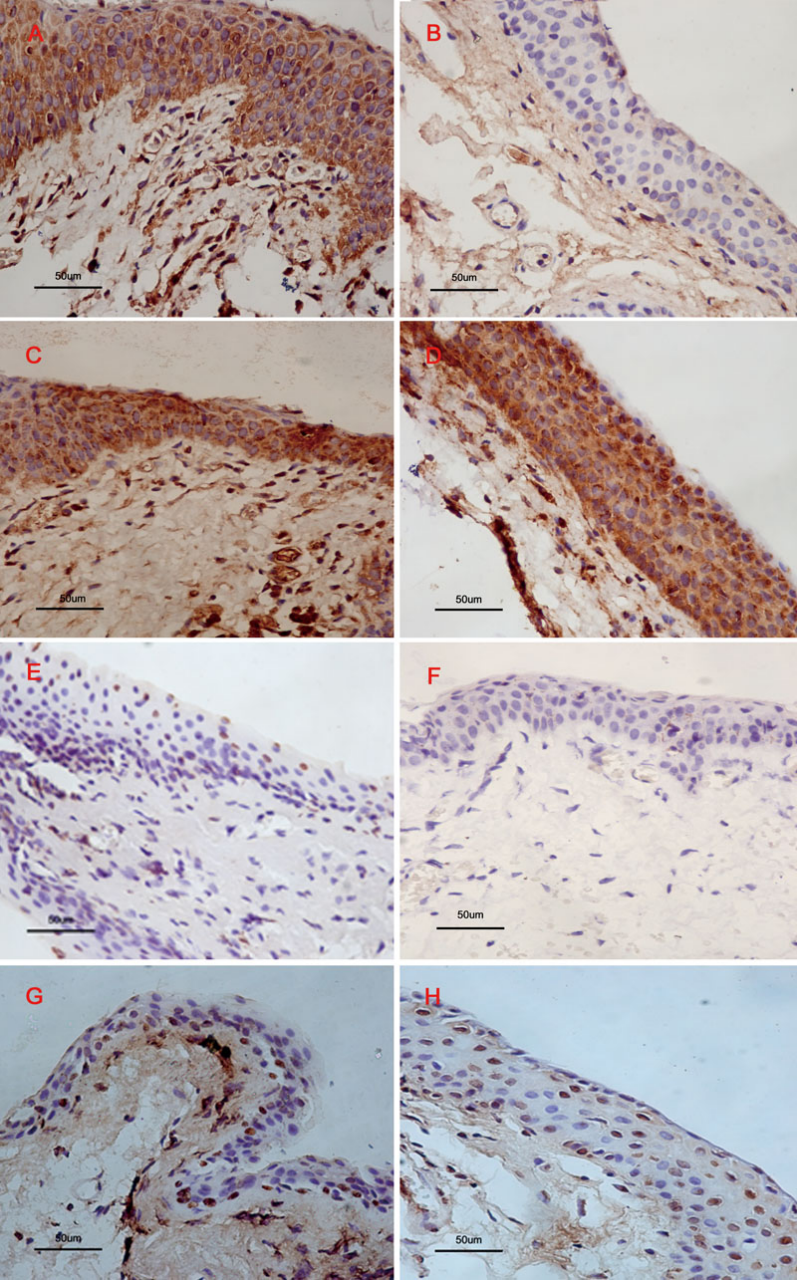
Immunohistochemical staining for Bcl-2, Bax, mutant p53, and TUNEL analysis positive cells in pterygium and normal conjunctiva samples. Human pterygium tissues (**A**, **C**, **E**, **G**); Human normal conjunctiva tissues (**B**, **D**, **F**, **H**). Bcl-2 showed strong expression in the cytoplasm throughout the entire width of the epithelial layer in pterygium (**A**). No expression was shown in the epithelial layer of Bcl-2 in normal conjunctiva (**B**). Bax showed strong expression in the cytoplasm throughout the entire width of the epithelial layer in both pterygium (**C**) and normal conjunctiva (**D**). mP53 immunostaining showed nuclear staining in the pterygium epithelial layer (**E**), but no expression in normal conjunctiva (**F**). For TUNEL analysis, the positive cells in pterygium showed nuclear staining in the basal layer of the epithelium (**G**). In normal conjunctiva, the positive cells showed nuclear staining in the whole width of the epithelial layer (**H**). All slides were counterstained with Mayer’s hematoxylin. Original magnifications: **A**-**H**, 400×.

The expression of mutant p53 was noted predominantly in the basal layer of epithelial cells. Mutant p53-positive staining was evaluated in 15 of the 33 cases of pterygia and in 0 of the 23 cases of normal conjunctive. The positive rate of mutant p53 was significantly higher in pterygia than in normal conjunctivas (p=0.001; Table 1, Figure 2E,F). Bcl-2- and Bax-positive staining were noted in the basal epithelial layer of cells. Bax showed a strong expression throughout the entire width of the epithelial layer in all of the pterygium and normal conjunctiva samples (Table 1, Figure 2C,D). Bcl-2 was expressed in the epithelial layer of all the pterygium samples and in two normal conjunctiva samples. The Bcl-2-positive rate was significantly higher in the pterygium samples (p=0.000, Table 1, Figure 2A,B). Caspase-3 was expressed in the epithelial layer in 14 of the 33 pterygium samples and 13 of the 23 normal conjunctiva samples. The positive rate of caspase-3 was not significantly different between the pterygium and normal conjunctiva samples (p=0.299, Table 1).

As age may be an important impact factor for apoptosis and proliferation in conjunctiva tissue, we analyzed the relationship between age and proliferation/apoptosis. The patients were divided into two groups (<70, ≥70), and the results showed that the positive rate of mP53 was significantly higher in the pterygia of the younger patients (Table 3). However, there was no difference in Capsase-3 or PCNA between the two age groups. With TUNEL analysis, in the epithelial layer, apoptotic cells were found mainly confined to the basal layer of the epithelial cells in pterygium samples (Figure 2H). In normal conjunctiva samples, the whole width of the epithelial layer expressed apoptotic cells (Figure 2G).

**Table 3:** Number of positive and negative results for cell proliferation and apoptosis biomarkers in different groups of pterygium samples according to age.

**Age**	**PCNA**		**Caspase-3**		**mP53**	
** **	**+**	**-**	**+**	**-**	**+**	**-**
<70	15	2	8	9	11	6
70–80	9	7	6	10	4	12
p value	0.095		0.579		0.022 *	

*p<0.05.

## Discussion

In this study, we tested the expression of two proliferation markers (PCNA and K_i_-67) and four apoptosis-related bio-markers (mP53, Bcl-2, Bax, and caspase-3) in pterygium and normal conjunctiva samples. The results showed that positive-PCNA, mP53, and Bcl-2 rates were significantly increased in the pterygium samples compared to normal conjunctiva samples. TUNEL analysis showed that the number of positive cells was lower in the epithelial layer of pterygia than that in normal conjunctivas. These results indicate that cell proliferation and apoptosis are involved in the pathogenesis of pterygia.

Pterygium, which invades the cornea forming a wing-like shape, is a proliferative, invasive, and highly vascularized tissue [14]. Previous studies have shown that appropriate cell apoptosis, not cell proliferation, plays an important role in the pathogenesis of pterygia [9,15]. However, studies regarding cell proliferation in the development of pterygia have also been reported [7,8,16]. The question was therefore raised whether proliferation or apoptosis participate in the pathogenesis of pterygia. This study was designed to clarify this issue.

In the bio-marker of proliferation, PCNA and K_i_-67 were widely studied, and we chose them as candidate markers to analyze. Proliferating cell nuclear antigen (PCNA) is a cofactor for DNA polymerase δ in both S phase and during DNA synthesis and is associated with DNA damage-repair mechanisms. Therefore, the expression of this molecule is useful for cell proliferation [17,18]. In the present study, PCNA was expressed in 24 (72.7%) of the 33 pterygium samples, but in only 7 (30.4%) of the 23 normal conjunctiva samples. The rates of positive PCNA were significantly higher in the pterygium samples than in the normal conjunctiva samples. This result is generally consistent with previous reports [7,19]. Because PCNA is just one of the important molecules that reflect the proliferation of pterygia, we further investigated K_i_-67 to test cell proliferation. Our results showed that K_i_-67 immunopositive cells in the epithelial layer of pterygia were significantly higher than in normal conjunctivas, which is the same result as in previous reports [13]. As PCNA and K_i_-67 are the important bio-markers of proliferation, our studies revealed that cell proliferation was involved in the pathogenesis of pterygia.

Although cell proliferation participates in the pathogenesis of pterygia, studies of apoptosis are also needed. We chose mutant p53, Bcl-2, Bax, and caspase-3 to analyze apoptosis. The mutation p53 protein may lead to fixing genome damage and to decreased cell cycle inhibition or decreased apoptosis [20]. Consistent with previous reports [10], we also found that the expression of mutant p53 was significantly increased in the epithelial layer of pterygium samples. These results suggest that the increased mutant p53 could possibly lead to decreased cell cycle inhibition or decreased apoptosis in the epithelial layer of the pterygium [21,22]. Bcl-2 proteins are involved in the response to apoptosis. Some of these proteins (such as Bcl-2 and Bcl-xl) are antiapoptotic, whereas others (such as Bak and Bax) are proapoptotic [23]. In our study, Bcl-2 was expressed in the epithelial layer of all the pterygium samples and two of the normal conjunctiva samples. Bax was expressed in all the pterygium and normal conjunctiva samples. It was not difficult to find from the expression of Bcl-2 and Bax that cell apoptosis in pterygia was weaker than in normal conjunctivas. If the balance of proapoptotic and antiapoptotic Bcl-2 proteins is disrupted, it will lead to the formation of the apoptosome and the activation of the caspases (such as caspases-3 and caspases-9) [24]. In this study, there was no significant difference in caspase-3 between normal conjunctivas and pterygia. Some reasons for this result are that caspase-3 may be not involved in the pathogenesis of pterygia and that the samples were selected from different sections of the pterygia. Apoptotic cells could be seen in the entire width of the epithelial layer in normal conjunctivas by TUNEL analysis. However, apoptotic cells were found mainly confined to the basal epithelial cells in pterygia; this finding is consistent with an earlier study [10]. Therefore, we can draw the conclusion that cell apoptosis participates in the development of pterygium.

As age is an important impact factor for proliferation and apoptosis in conjunctiva tissue, we divided the samples into two groups according to age (<70, ≥70). The results showed that the positive rate of mP53 was significantly increased in pterygia in the younger patients. mP53 protein decreased cell cycle inhibition and apoptosis, which may be the reason that pterygium relapses more easily in younger patients.

There were some limitations in our study. The best way to determine whether caspase-3 is involved in the pathogenesis of pterygia is to measure pro-caspase 3 and active caspase-3, which we intend to do in a further study. In conclusion, our study suggests that both cell apoptosis and cell proliferation processes are strongly associated with the development and progression of pterygia. The mechanisms of cell apoptosis and cell proliferation in pterygium are complicated, and further study is needed.
